# Nematode microRNAs can Individually Regulate Interferon Regulatory Factor 4 and mTOR in Differentiating T Helper 2 Lymphocytes and Modulate Cytokine Production in Macrophages

**DOI:** 10.3389/fmolb.2022.909312

**Published:** 2022-06-28

**Authors:** Julien Soichot, Nathalie Guttmann, Hubert Rehrauer, Nicole Joller, Lucienne Tritten

**Affiliations:** ^1^ Institute of Parasitology, Vetsuisse Faculty, University of Zurich, Zurich, Switzerland; ^2^ Functional Genomics Center Zurich, ETH Zurich/University of Zurich, Zurich, Switzerland; ^3^ Department of Quantitative Biomedicine, University of Zurich, Zurich, Switzerland

**Keywords:** parasitic nematode, microRNA, immunomodulation, T cells, macrophages, IRF4, mTOR, cytokines

## Abstract

Parasitic nematodes are masterful immunomodulators. This class of pathogens has evolved a spectrum of sophisticated strategies to regulate and evade host immune responses, mediated through the release of various molecules. In this context, the release of microRNAs (miRNAs), short post-transcriptional regulators of gene expression, has been of particular interest in the host-parasite interplay. Evidence that parasite-derived miRNAs modulate host innate and adaptive immune responses has become increasingly compelling. However, since miRNAs are usually contained in extracellular vesicles containing other mediators, it is difficult to assign an observed effect on host cells to miRNAs specifically. Here, the effects of some abundantly secreted miRNAs by nematodes used as models of gastrointestinal infections (*Heligmosomoides polygyrus bakeri*, *Trichuris muris* and *Ascaris suum*) were evaluated, addressing the potential of parasite miRNAs to impair *in vitro* differentiation of two important types of immune cells in the context of helminth infections, Th2 lymphocytes and macrophages. Mimicking a continuous exposure to low concentrations of nematode miRNAs, the interferon gamma signaling, the IL-2/STAT5 signaling, and the mTOR signaling pathways were identified as downregulated by Hpo-miR-71-5p. Interferon regulatory factor 4 (*Irf4*) was validated as a target of Hpo-miR-71-5p, while *Mtor* is targeted by Asu-miR-791-3p, abundant in the *T. muris* secretions. By trend, Hpo-miR-71-5p impacts mildly but consistently on the amounts of inflammatory cytokines in unpolarized macrophages but leads to slightly increased IL-10 level in alternatively activated cells. In addition, our data suggests that transfected miRNAs remain for days in recipient cells, and that Hpo-miR-71-5p can incorporate into mouse Argonaute protein complexes. Nematode miRNAs can impair both innate and adaptive arms of host immunity*.* Hpo-miR-71-5p in particular, absent in mammals, interacts with host genes and pathways with crucial involvement in anthelmintic immune responses. This report brings new insights into the dynamics of miRNA-driven immunomodulation and highlights putative targeted pathways. Although the absolute repression is subtle, it is expected that the dozens of different miRNAs released by nematodes may have a synergistic effect on surrounding host cells.

## Introduction

Parasitic helminths are macroscopic pathogens of plants and animals, including humans. In 2013, over 2 billion human cases were attributed to the main helminth species (Herricks et al., 2017). In 2020, 24% of the world’s population was infected with soil-transmitted helminths (*Ascaris lumbricoides*, *Trichuris trichiura*, and the hookworms *Necator americanus* and *Ancylostoma duodenale*), affecting primarily the poorest and most deprived populations of tropical and subtropical areas of the world (World Health Organization, 2020). However, helminth infections cause not only diseases of the poor but also have a colossal economic impact in Western countries, especially in the livestock industry. Gastrointestinal (GI) nematodes alone cause production losses in up to 50% of ruminant farms analyzed in several European studies (Charlier et al., 2014). Improvements in diagnostics and control interventions will be crucial, as the development of drug resistance has been fast and severe, for the most part in livestock parasites (Kaplan and Vidyashankar, 2012; Selzer and Epe, 2020).

Upon host colonization, helminth infections and resulting diseases tend to become stable and chronic. This is at least in part due to the exceptional abilities of this class of pathogens to manipulate their hosts and modify surrounding tissues to their own advantage (Maizels and McSorley, 2016).

The so-called “modified type 2 immunity” elicited by helminths in their mammalian hosts is characterized by a CD4^+^ T helper 2 (Th2)-driven response, accompanied by regulatory T cell (Treg) subsets that dampen inflammation (Allen and Maizels, 2011; Girgis et al., 2013). Th2 responses confer partial resistance to parasitic worms and help control infection and inflammation while promoting wound healing. While hosts mount immune responses to constrain the detrimental impact of such infections, helminths have evolved a spectrum of sophisticated strategies to regulate and evade host immune responses. As a result, the immune pathways that would be engaged to lead to worm expulsion are neutralized, and immunity is globally dampened, also affecting responses to unrelated bystander antigens (Maizels and McSorley, 2016). These effects are mediated through the release of parasite-derived molecules of various types.

Excretory/secretory (E/S) molecules of the phylum *Nematoda* have been studied extensively for their immunomodulatory properties. E/S soluble proteins in particular affect host immune responses through various mechanisms from mimicking host molecules, to inhibiting immune processes, degrading key host molecules, or facilitating entry into and movement within hosts (Bellafiore et al., 2008; Hewitson et al., 2009; Shepherd et al., 2015; Tritten et al., 2021). Other types of molecules contribute to the continuous dialogue between helminth and host, including glycans, lipids (Whitehead et al., 2020) and nucleic acids (Buck et al., 2014; Sotillo et al., 2020). Following pioneering studies demonstrating host gene modulatory effects of nematode microRNAs (miRNAs) (Buck et al., 2014), non-coding RNAs have been of particular interest in the host-parasite interplay. miRNAs are short post-transcriptional regulators of gene expression that are integral to virtually all biological processes in eukaryotes. Silencing of the partially complementary mRNA targets occurs through a combination of translational repression and/or mRNA destabilization, ultimately leading to mRNA degradation (Jonas and Izaurralde, 2015). For their regulatory functions, miRNAs associate with protein complexes including Argonaute proteins (AGO), part of the RNA induced silencing complex. The way a targeted mRNA becomes silenced depends on the degree of complementarity between miRNA and the mRNA site. Full complementarity enables catalytically active AGO to cleave the mRNA directly, while incompletely complementary miRNAs require recruitment of additional protein partners to mediate silencing through a combination of repression, deadenylation, decapping, and degradation of the transcript (Jonas and Izaurralde, 2015). miRNA-driven repression is characterized by both redundancy and pleiotropy in targeted genes and pathways. Evidence that parasite-derived miRNAs modulate host innate and adaptive immune responses has become increasingly compelling (Coakley et al., 2015, 2016). Available data suggest a widespread incorporation of helminth miRNAs into a broad range of host cells. Various crucial host immune functions are repressed by nematode miRNAs *in vitro* following internalization of parasite-derived extracellular vesicles (EVs), as well as *in vivo* (Buck et al., 2014; Zamanian et al., 2015; Coakley et al., 2017; Eichenberger et al., 2018a; Liu et al., 2019; Meningher et al., 2020; Tran et al., 2021). miRNAs released from *Litomosoides sigmodontis* were preferentially detected in host macrophages *in vivo* (Quintana et al., 2019), similar to miRNAs from *Schistosoma japonicum* EVs (Liu et al., 2019). miRNAs found in *S. japonicum* EVs regulated host macrophage functions and are able to incorporate into mouse AGO2 *in vitro* (Liu et al., 2019). *Schistosoma mansoni* EV-contained miRNAs are taken up by host T helper cells and impair Th2 differentiation by targeting MAP3K7 and inhibiting NF-κB activity. The same paper reported the presence of *S. mansoni* miRNAs in Peyer’s patches and mesenteric lymph nodes of infected hosts (Meningher et al., 2020). Similarly, *Haemonchus contortus*-derived circulating miRNAs were detected in infected sheep lymph nodes (Gu et al., 2017). The trematode *Fasciola hepatica* hijacks host miRNA machinery in macrophages, where they negatively regulate production of inflammatory cytokines and modulate early immune response to the parasite (Tran et al., 2021). Several gaps remain in our understanding of host gene repression by helminth miRNAs (Claycomb et al., 2017). Currently, it seems that in order to regulate host genes, helminth miRNAs must be loaded onto host AGO2 proteins, taking advantage of the effector machinery in place (Donnelly and Tran, 2021), an interaction likely dictated by miRNA sequence and structure, and perhaps also by cell-specific needs (Ricafrente et al., 2020; Brosnan et al., 2021; Donnelly and Tran, 2021; Luo et al., 2021).

In addition to cells of the innate immune system such as macrophages, important recipient cells of nematode miRNAs (Quintana et al., 2019), a few reports indicate that T lymphocyte development and differentiation may be commonly targeted by helminth-derived miRNAs (Duguet et al., 2020; Meningher et al., 2020). Here, we studied the effects of some abundantly secreted miRNAs by nematodes used as models of gastrointestinal infections (*Heligmosomoides polygyrus bakeri*, *Trichuris muris* and *Ascaris suum*). Computational target predictions in the mouse genome identified the master transcription factor of Th2 differentiation *Gata3* among other important genes. We addressed the potential of parasite miRNAs to impair Th2 and macrophage differentiation *in vitro*, which evolved as a response to helminth infections and confer some degree of anthelmintic resistance (Allen and Maizels, 2011). We showed that crucial pathways for the differentiation into Th2 cells are downregulated by transfection of synthetic nematode miRNAs and validated interactions with some of their predicted targets. By trend, macrophages exposed to nematode miRNAs produced reduced amounts of inflammatory cytokines. We conclude that nematode miRNAs can impair both the innate and adaptive arms of host immunity by interacting with essential genes.

## Materials and Methods

### Bioinformatics Analysis

Secreted miRNA lists from *H. polygyrus bakeri*, *T. muris*, and *A. suum* were retrieved from the literature (Buck et al., 2014; Tritten et al., 2017; Eichenberger et al., 2018b; Hansen et al., 2019). Target prediction in the mouse genome for the top 20 most abundant miRNAs was performed as described previously (Duguet et al., 2020) using the TargetScan software package v. 7 (http://www.targetscan.org), implemented as a standalone workflow under iPortal (Kunszt et al., 2015) and openBIS (Bauch et al., 2011), using default parameters (Agarwal et al., 2015). The model takes 14 different features of the miRNA, miRNA site, or mRNA into account to predict which sites within mRNAs are most effectively targeted by miRNAs and further relies on a multi-species alignment of 3′UTR across 84 vertebrate species derived from the UCSC genome browser. Results were deposited in Mendeley Data: https://data.mendeley.com//datasets/358nm9mhpr/1). Predicted targets were screened using R Studio (4.0.3 (R Core Team, 2017)) against lists of typical gene markers and molecules involved in development of Th2 cells (Stubbington et al., 2015; Zhu, 2015), as well as all mouse genes listed in the 15 KEGG pathways under 5.1. “Immune system” (Kanehisa et al., 2017) accessed in May-June 2018. KEGG pathways comprised: Hematopoietic cell lineage, Toll-like receptor signaling pathway, NOD-like receptor signaling pathway, C-type lectin receptor signaling pathway, Natural killer cell mediated cytotoxicity, Antigen processing and presentation, T cell receptor signaling, Th1 and Th2 cell differentiation, Th17 cell differentiation, IL-17 signaling pathway, Fc epsilon RI signaling pathway, Fc gamma R-mediated phagocytosis, Leukocyte transendothelial migration, Intestinal immune network for IgA production, and Chemokine signaling pathway.

### T Cell Differentiation

Wild-type female C57BL/6J mice were ordered from Envigo (Netherlands) and euthanized with CO_2_ between 5 and 12 weeks of age to isolate naïve CD4^+^ T cells. The spleen and the inguinal, axillary, brachial, and cervical lymph nodes were dissected and processed as described (Flaherty and Reynolds, 2015), with some modifications. Under sterile conditions, tissues were crushed against a 100 μm cell strainer (Corning) and filtered through a second 100 μm cell strainer. Cells were pelleted at 475 x *g* for 5 min at 4°C. Cells from lymph nodes were resuspended in sterile PBS and kept on ice. Splenic cells were resuspended in 1 ml ACK lysis buffer (Gibco) for 1 min to remove erythrocytes and washed twice with PBS.

Naïve CD4^+^ T cells were purified by depletion of memory CD4^+^ T cells and non-CD4^+^ T cells using a mouse Naïve CD4^+^ T cell Isolation Kit (Miltenyi Biotec) on LS columns (Miltenyi Biotec), following the manufacturer’s instructions. Isolated naïve CD4^+^ T cells were counted and cultured in anti-CD3ε (clone 145-2C11, eBioscience) and α-CD28 (clone 37.51, eBioscience) coated plates in 500 µl RPMI complete (RPMI-1640 containing 10% v/v FBS (Corning), 100 U/ml penicillin, 100 μg/ml streptomycin, 2 mM l-glutamine and 50 μM β-mercaptoethanol), supplemented with 10 ng/ml recombinant mouse IL-4 (Biolegend), 30 U/ml recombinant human IL-2 (Biolegend), and 5 μg/ml anti-IFN-γ (clone XMG1.2, eBioscience), and 2 μg/ml anti-CD28 (Flaherty and Reynolds, 2015)). Cells were cultured at 37°C, 5% CO_2_. After 48 h, 500 µl fresh RPMI complete were added to each well. Cells were counted at 72 h and seeded at 1 × 10^6^ cells/ml in RPMI complete containing only 20 U/ml recombinant human IL-2 (Biolegend) and 5 μg/ml anti-mouse IFN-γ (clone XMG1.2, eBioscience). After 92 h in culture, cells were re-stimulated at 1 × 10^6^ cells/ml with 1 μg/ml anti-CD3ε (clone 145-2C11, eBioscience) for 4 h prior to RNA extraction, or with 1 μg/ml ionomycin (Merck) and 10 ng/ml PMA (Sigma-Aldrich) for 4 h with the addition of 3 μg/ml brefeldin A (ThermoFisher Scientific) for the last 3 h for FACS analysis, as described (Flaherty and Reynolds, 2015). Total RNA was extracted using an RNeasy Plus mini kit (Qiagen). RNA concentration was quantified using a Qubit 4 Fluorometer. Prior to FACS analysis, cells were treated with 10 μg/ml brefeldin A and with a dilution of 1:1,000 Live/Dead Fixable Near-IR Dead Cell Stain Kit (Invitrogen) according to the manufacturer’s instructions. Cells were fixed and permeabilized with a BD Cytofix/Cytoperm kit (BD Biosciences) and intracellular staining was carried out with APC anti-mouse IFN-γ antibody (Biolegend) diluted 1:20 and PE-efluor610 anti-mouse IL-13 antibody (Invitrogen) diluted 1:100. Data were acquired on a Cytoflex S flow cytometer (Beckman Coulter) and further analyzed in FlowJo Software v10.8. Compensation matrix was created using VersaComp Antibody Capture Bead Kit (Beckman Coulter).

### Differentiating T Cell miRNA Transfection

Cells were transfected with synthetic miRNAs once or several times at various time points, between 0 and 90 h after culture start. We used mirVana miRNA mimics (Ambion, LifeTechnologies) of Hpo-miR-71-5p (mature sequence: UGA​AAG​ACA​UGG​GUA​GUG​AGA​C), Hpo-miR-100-5p (mature sequence: AAC​CCG​UAG​AUC​CGA​ACU​UGU​GU), and Hpo-miR-10021-5p (mature sequence: UGA​GAU​CAU​CAC​CAU​AAG​CAC​A), as they are among the most abundant freely circulating miRNAs found in *H. polygyrus bakeri* culture supernatants (Buck et al., 2014; Tritten et al., 2017). We used additionally Hpo-miR-1-3p (mature sequence: UGG​AAU​GUA​AAG​AAG​UAU​GUA) the mirVana miRNA mimic universal negative control #1 (Ambion, LifeTechnologies), a random sequence miRNA mimic molecule validated to not produce identifiable effects on known miRNA function in human cells. miRNA mimics were transfected into 2 × 10^5^—1 × 10^6^ cells using lipofectamine RNAiMax (Invitrogen) and OPTI-MEM I 1x (Gibco) following the manufacturer’s protocol at final concentrations of 10 nM or 50 nM. A combination of miR-71, miR-10021, and miR-100 was also tested at the same total concentration (e.g., 3.33 nM of each individual miRNA). Information on miRNAs used in this work is provided in more detail in Supplementary Table S4.

### Transfection Efficiency and Imaging

The proportion of transfected Th2 cells was determined by FACS analysis following transfection with 50 nM BLOCK-iT fluorescent oligo (Invitrogen) and lipofectamine RNAiMax (Invitrogen) as described above. The proportion of FITC-labeled cells was determined 1–48 h post-transfection in cells at various stages throughout the differentiation protocol (from naïve CD4^+^ T cells to 96 h in culture) exposed to i) BLOCK-iT fluorescent oligo + lipofectamine, ii) BLOCK-iT fluorescent oligo without lipofectamine and iii) lipofectamine only. Cells transfected with 40 nM BLOCK-iT fluorescent oligo were imaged 1 and 3 h post-transfection and co-stained with LysoTracker Red DND-99 (Invitrogen) for live cell imaging in a Leica DMI6000 B inverted fluorescence microscope (Leica Microsystems, Germany) equipped with a Leica DFC365 FX camera (Leica) and recorded using the LAS X software (Leica). Images were compiled in ImageJ (v.1.53k).

### Transcriptome Analysis

Total RNA from 3 independent experiments of differentiating Th2 cells treated every 24 h with 10 nM miRNA was quality controlled using the fragment analyzer (Agilent Technologies, Santa Clara, CA, United States) at the Functional Genomics Center Zurich. Subsequent library preparation and sequencing was performed using Illumina Truseq mRNA library protocol. RNA sequencing was performed using an Illumina Novaseq 6000 sequencer with single read 100bp. Sequencing reads were aligned using STAR (Spliced Transcripts Alignment to a Reference) (Dobin et al., 2013) with the Ensembl mouse genome build GRCm38.p6 (provided by GENCODE M23 release) as reference. Gene expression values were obtained with the function featureCounts from the R package Rsubread (Liao et al., 2013). Differential expressed (DE) genes analysis was computed with the Bioconductor package EdgeR. Determination of cell types and their proportion in our samples was assessed by Digital Sorting Algorithm (Zhong et al., 2013). Following the analysis of the estimated cell type fractions, DE genes were computed comparing different miRNA treatments versus the universal negative control, or versus lipofectamine (the transfection reagent) only, using the donor mouse as experimental factor. Variability caused by differences in cell type composition was estimated and modeled using the RUVSeq package (Risso et al., 2014). Raw data was deposited on GEO https://www.ncbi.nlm.nih.gov/geo/query/acc.cgi?acc=GSE180829 (reviewer token: qrchacisjnojnmv) DE genes (cutoff: *p* < 0.01) were subjected to pathway analysis in Enrichr (Kuleshov et al., 2016; Xie et al., 2021), using the MSigDB Hallmark 2020 as library (Liberzon et al., 2015).

### Macrophage Assays–miRNA Transfections and Polarization

Raw 264.7 blood macrophages (cell line 91062702, ECACC) were seeded at a density of 5 × 10^5^ cells in 500 µl DMEM 1x (Gibco) containing 10% v/v FBS (Corning), 100 U/ml penicillin, and 100 μg/ml streptomycin, and transfected with 100 nM mirVana mimics (list) and lipofectamine RNAiMax (Invitrogen). After 24 h, cells were collected, washed with PBS and resuspended in 500 µl fresh media without further addition (M0: neutral activation status), or polarized with 20 ng/ml IFN-γ (Biolegend) and 100 ng/ml lipopolysaccharide (eBioscience; M1: classical activation), or 10 ng/ml recombinant mouse IL-4 (Biolegend; M2: alternative activation). Cells were incubated for 6 h at 37°C, 5% CO_2_, and collected for RNA isolation. Supernatants were centrifuged for 10 min at 1,500 x *g*, 4°C to remove debris and frozen at -80°C. The experiment was conducted 3 times independently.

### Luminex Assay–Cytokines in Supernatants

Supernatants from Raw 264.7 macrophages were analyzed on a Luminex FlexMAP 3D using a Milliplex MAP Mouse Cytokine/Chemokine Magnetic kit (MCYTMAG-70K-PX32; Merck Millipore) for the simultaneous measurement of 32 cytokines and chemokines, following the manufacturer’s recommendations. Median fluorescent intensity data were analyzed in GraphPad Prism (v. 9.2.0) using a five-parameter logistic curve-fitting method for calculating cytokine/chemokine concentration as recommended by the manufacturer. Data were produced for 3 independent experimental replicates. Concentrations and the Dunnett’s test were computed in RStudio.

### Reporter Assays

Predicted target sites were inserted on the pmirGLO dual luciferase miRNA target expression vector (Promega) and cloned into HeLa cells. Briefly, 20,000 cells were seeded into Costar flat-bottom white 96-well plates in 200 µl media (DMEM 1x high-glucose (Gibco) containing 10% v/v FBS (Corning), 100 U/ml penicillin and 100 μg/ml streptomycin) and allowed to attach for 24 h at 37°C, 5% CO_2_. HeLa cells were co-transfected with 200 ng/well vector (with or without target site insert) and different miRNA concentrations (50, 25, or 0 nM) using lipofectamine reagent 3000 (Invitrogen), following manufacturer’s instructions. Cells were lysed and luminescence was quantified sequentially (Firefly followed by Renilla) using the Dual-Glo luciferase assay system (Promega). Assays were conducted 3 times independently, with 5-6 well per condition. A two-step normalization analysis was undertaken to quantify luciferase relative activity (Campos-Melo et al., 2014). Dunnett’s tests were performed for each miRNA concentrations separately, with the luminescence ratios obtained on the vector containing the insert versus those obtained from the empty vector, as described (Campos-Melo et al., 2014).

### Mouse AGO2 Pull Down Assays and RT-qPCR

To verify that synthetic miRNAs were incorporated into host cell silencing machinery, differentiating mouse Th2 cells were transfected at 92 h with 75 nM mirVana mimic Hpo-miR-71-5p, which is a nematode-specific miRNA, absent in mammalian cells. Mouse AGO2 pull-down assays followed by miRNA isolation were conducted 4 h later using a microRNA Isolation kit Mouse Ago2 (Fujifilm). For each condition (Hpo-miR-71-5p transfected and untransfected samples), 4 × 10^6^ cells were used in 3 biological replicates representing different donor mice. The same procedure was applied to fresh mesenteric lymph nodes from 2 *H. polygyrus bakeri* infected mice. A custom-designed stem-loop RT-qPCR assay (modified from (Tritten et al., 2014); stem-loop primer for RT: 5′-CCA GTG CAG GGT CCG AGG TA-3′, forward primer: 5′-TCG GGT AGT GAA AGA CAT GGG TAG T-3′, universal reverse primer: 5′-CCA GTG CAG GGT CCG AGG TA-3′, probe: 5′- FAM - CGC ACT GGA TAC GAC GTC TC- MGBQ5 - 3′) was employed to detect the presence of transfected Hpo-miR-71-5p in miRNAs isolated from mouse Ago2 pull-downs. We used the RT Maxima H Minus reverse transcriptase (Thermo Scientific) and the TaqMan Fast Advanced master mix (Applied Biosystems); amplification was performed on a QuantStudio 7 Flex real-time PCR system (Applied Biosystems/Life Technologies).

### Ethics Approval

Ethical clearance for work involving mice was obtained from the Cantonal Veterinary Office of Zurich (permit number ZH186/18). All experiments were conducted in accordance with the Swiss cantonal and national regulations on animal experimentation. The *H. polygyrus bakeri* life cycle is maintained by Prof. J. Keiser at the Swiss TPH (University of Basel), under license 2070 granted by the Cantonal Veterinary Office of Basel-Stadt.

## Results

**FIGURE 1:**
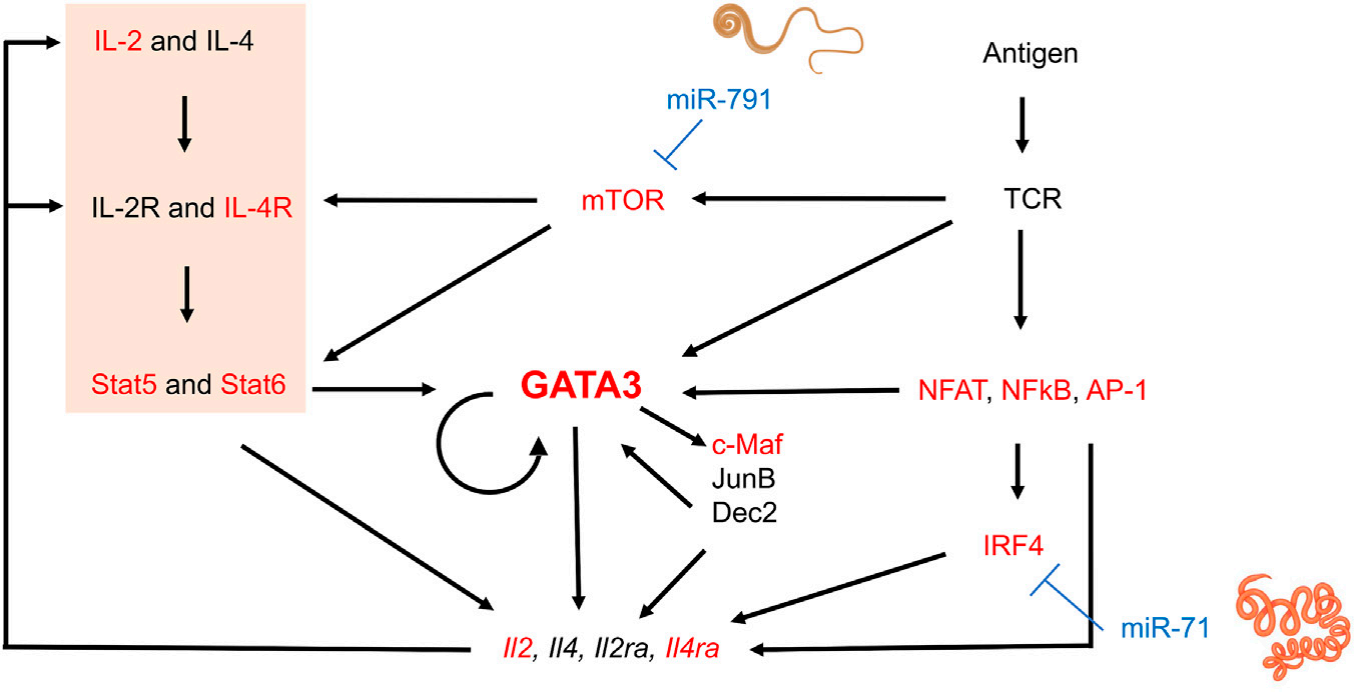
Schematic transcriptional network and positive regulation during Th2 lymphocyte differentiation and interference by nematode miRNAs. Elements in red represent genes of which at least some isoforms represent predicted targets of the 20 most abundantly secreted *H. polygyrus bakeri, T. muris* and *A. suum* miRNAs. Briefly, T cell receptor (TCR) stimulation activates downstream molecules such as nuclear factor of activated T-cells (NFAT), nuclear factor-κB (NFκB), and AP-1, in turn leading to upregulation of interferon regulatory factor 4 (IRF4) (Zhu, 2015). IL-4-mediated Stat6 activation and other signaling pathways also induce GATA3 expression. GATA3 may directly or indirectly regulate Th2 cytokine expression by inducing other transcription factors such as c-Maf, JunB, or Dec2, some of which may further sustain GATA3 expression. The IL-2—Stat5 axis is also crucial for Th2 cytokine production and was significantly downregulated by our transfected miRNAs (orange block). Activated T cells produce IL-2 and IL-4, among others, upregulating IL-2 and IL-4 receptors, creating a positive feedback loop. mTOR in the complex mTORC2 is required for Th2 differentiation *via* two mechanisms: i) repression of expression of negative regulators of IL4-R and STAT6, and ii) promotion of NF-κB-mediated transcription (Chi, 2012). Some elements of this image were created with Biorender.com

Hypothesizing that nematode miRNAs present at higher concentrations may have a greater impact on host gene expression, we focused our analysis arbitrarily on the 20 miRNAs most abundantly secreted by three parasitic species, *H. polygyrus bakeri*, *T. muris* and *A. suum* (Supplementary Table S1). Genes resulting from target predictions of these miRNAs were matched to genes functionally relevant for T cells and macrophages. The number of predicted interactions between miRNAs and each gene—regardless of the nature of the predicted miRNA binding site and without taking overlapping binding sites into consideration—highlights putative differences in miRNA-mediated host gene regulation by the three nematodes (Supplementary Table S2). For instance, *Gata3* is predicted to be targeted 5x by *H. polygyrus bakeri*, 1x by *T. muris*, and 8x by *A. suum* miRNAs. Guided by the transcriptional network and positive feedback regulation during Th2 cell differentiation (summarized in (Yang et al., 2013; Zhu, 2015), Figure 1), we functionally validated several predicted targets of interest in differentiating Th2 cells. Several key elements of the transcriptional network for Th2 differentiation represent computationally predicted targets (Figure 1). Among them were genes encoding the Th2 master regulator GATA3 and the IL-4Rα, another molecule central to Th2 immunity elicited against helminths (Allen and Maizels, 2011). Also, the mechanistic target of rapamycin (mTOR) was among the predicted gene targets of *T. muris* secreted miRNAs. The most frequently targeted genes by *H. polygyrus bakeri* and *A. suum* abundantly secreted miRNAs: *D1ertd622e* (or macrophage immunometabolism regulator (MACIR)), predicted to be targeted 169x and 162x, respectively, followed by cAMP response element modulator (*Crem* 113x and 119x, respectively), and *Mapk10*, (84x and 85x, respectively). miRNAs secreted by *T. muris* were predicted to target *D1ertd622e* 191x, *Crem* 113x, and catenin delta 1 (*Ctnnd1*), 79x (Supplementary Table S2).

### Individual Abundant Nematode miRNAs Enter Host Cells but do Not Abrogate Th2 Differentiation or Proliferation

**FIGURE 2:**
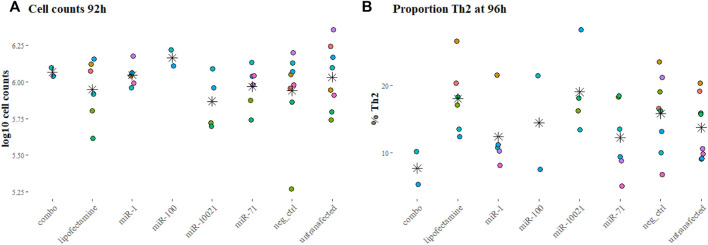
Cell counts and proportions of fully differentiated Th2 cells. Cell counts after 92 h into culture **(A)** and proportion of Th2 cells at the end of the experiments (96 h, **(B)**, as reflection of IL-13 positive labeling, measured by FACS. Colors indicate independent experiments; stars indicate the mean for each miRNA treatment. Cell counts are expressed in log_10_ of average cell counts.

To reproduce the regular or constant exposure to worm secretions, as expected during an ongoing infection, the impact of daily transfections with synthetic nematode miRNA on cell proliferation was monitored at 92 h in several experiments (two to eight replicated per condition). Cell counts were not significantly different across miRNA treatments or controls (*p* = 0.58, Figure 2A).

The proportion of Th2 cells, measured by flow cytometry at 96 h and defined by expression of IL-13, varied markedly across experiments (Figure 2B). This was also observed with cells from different individual mice of the same batch (age and sex matched, but not necessarily litter mates). On average per transfection condition, only 7.66% (±3.45%; based on 2 experiments) of cells exposed to the 3-miRNA combination (combo; miR-71 + miR-100 + miR-10021) expressed IL-13; 12.23% (±5.45% miR-71) and 19.0% (±6.49%; miR-10021) of cells were considered fully differentiated Th2. Untransfected cells reached on average 13.69% (±4.62%) of Th2; cells exposed to the universal negative control (UNC) showed on average 15.79% (±5.64%) differentiated Th2 cells (Figure 2B). Th2 proportions across treatments and controls were not significantly different (*p* = 0.20). The remaining cells were most likely not fully differentiated or uncommitted as *in vitro* differentiation procedures usually yield a maximum of 25% of Th2 cells (Flaherty and Reynolds, 2015). Cells expressing IFN-γ were in the range of 1% of the population or below, confirming a clean Th2 culture.

**FIGURE 3:**
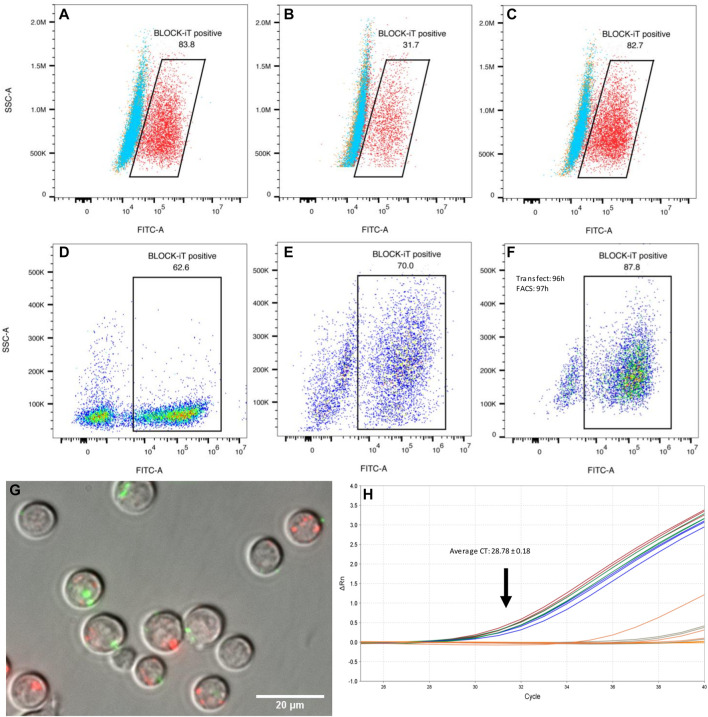
Transfection efficiency. **(A–C)** Raw 264.7 macrophages (mϕ) transfected with a fluorescent oligonucleotide (representative examples). **(A)** 83.8% of unpolarized macrophages showed FITC fluorescence 1 h post transfection with BLOCK-iT fluorescent oligonucleotide (FITC labeled) and lipofectamine (red). Cells exposed to the oligo only are shown in blue, or to the transfection reagent only, in orange. **(B)** Classically activated macrophages (M1) showed only 31.7% transfection efficiency with the fluorescent oligo and lipofectamine (red). **(C)** Alternatively activated macrophages (M2) showed a high transfection efficiency, similar to M0 cells, with 82.7% cells showing FITC labeling. Transfection efficiency in differentiating Th2 cells **(D–F)**. CD4^+^ naïve T cells were transfected with BLOCK-iT fluorescent oligo upon start of the differentiation protocol (day 0). Transfection efficiency was measured after 1 h **(D)** and again after 48 h **(E)**. Cells transfected at the end of the differentiation protocol (96 h) showed 87.8% of FITC-labelled cells when the oligo was introduced into cells with lipofectamine **(F)**. **(G)** Live cell imaging of transfected differentiating Th2 cells. CD4^+^ T cells were transfected at 96 h of Th2 differentiation with a FITC-labelled oligo (green) and imaged 1 h post-transfection (magnification: 60 x). Lysosomes were stained with Lyso-Tracker red DND-99 (red). There is a priori no co-localization between introduced oligonucleotides and lysosomes. **(H)** Real-time PCR amplification of Hpo-miR-71-5p from miRNA following mouse AGO pull-downs, 4 h after transfection of Th2 cells with miRNA mimics. miRNA transfected samples are represented by blue, red, and green replicate curves.

Transfection efficiency in macrophages and differentiating Th2 lymphocytes was assessed with a fluorescently labeled oligonucleotide (Figure 3). Unpolarized and alternatively activated macrophages (M2) showed a high rate of oligo uptake (>80%), only when the transfection reagent was used. In contrast, M1 macrophages showed a lower transfection efficiency, with a third of cells displaying fluorescence. Regardless of time in culture and differentiation status, at least 60% of live CD4^+^ T cells showed detectable signal by FACS analysis, as early as 1 h post-exposure to lipofectamine-oligonucleotide complexes. The signal was still detectable in 64.8 and 70% of cells 48 h post-transfection (2 samples, one of them shown in Figures 3D,E). A maximum of 10% of cells exposed to the oligonucleotide only (without transfection reagent) showed staining. Live cell imaging 1 h post-transfection allowed the detection of FITC-labeled oligonucleotide in small clusters, which did not co-localize with lysosomes and other acidic organelles (Figure 3G). However, this does not exclude the possibility that diffusion of oligonucleotides into the cytosol may have occurred. Lipofectamine transfection reagents are typically expected to deliver their contents *via* fusion with the cell membrane, rather than by active endocytosis, thereby releasing their cargo into the cytosol.

### Nematode miR-71 Incorporates Into AGO2 Protein Complexes in Transfected Differentiating Th2 Cells to Regulate Mouse Gene Expression

Our understanding of host gene repression by helminth miRNAs remains incomplete. A currently popular concept is that helminth miRNAs must be loaded onto host silencing protein complexes (AGO2 in particular) to regulate host genes (Donnelly and Tran 2021). This interaction is likely to be at least partially dictated by miRNA sequence and structure (Luo et al., 2021). Because miR-71 is specifically expressed by nematodes and does not have a close homolog in mammalian systems, we asked whether it would also be able to interact with host AGO2. Differentiating Th2 cells (after 92 h in culture) were transfected with miR-71; cells were lysed for AGO2 precipitation after 4 h, followed by miRNA isolation. As expected, the mature Hpo-miR-71-5p was detected by RT-qPCR (Average CT = 28.78 ± 0.18) in transfected cells but not detected in untransfected cells (CT > 37; Figure 3H). However, using the same real-time PCR assay, miR-71 was not detected in the mesenteric lymph nodes from *H. polygyrus bakeri* infected mice, likely present below the detection capacity of the assay.

### Nematode miRNAs Downregulate Critical Genes and Pathways of the Th2 Differentiation Process

Throughout the 4-day differentiation, CD4^+^ cells were transfected daily with low doses (10 nM) of the three most abundant *H. polygyrus bakeri* secreted miRNAs, to reproduce the expected constant pressure exerted by nematode miRNAs on surrounding host cells. A whole transcriptome analysis was carried out with total RNA collected after 4 days in culture. Sequenced transcripts from three independent differentiation-transfection experiments resulting from a whole transcriptome analysis displayed strong batch effects and inter-individual variability across donor mice. To correct for non-specific responses upon introduction of nucleic acids into cells, all pairwise comparisons were made against treatment with UNC, for which no target is known. Only between 11 and 110 genes were DE across comparisons between synthetic miRNA treatments and the UNC miRNA, cutoff: *p* < 0.01, across all fold-changes and FDRs (Table 1, Supplementary Table S3). No upregulated pathways appeared below the *p*-value threshold of 0.05, except in the comparison between the transfection reagent without miRNA and the UNC. The upregulation of these eight pathways can be interpreted as non*-*specifically downregulated by the UNC miRNA. Some of these overlap with pathways downregulated by other miRNAs, however, without overlap of the contributing genes.

**TABLE 1:** Most strongly up- and downregulated pathways. DE genes (p < 0.01) were submitted to Enrichr for pathway enrichment against the MSigDB Hallmark 2020 library. Combo: combination of miR-100, miR-10021, and miR-71 in equal parts, where the total amount of miRNA introduced is the same as for individual miRNAs (i.e., 10 nM). UNC: universal negative control. Data for miR-100, miR1 and combo are from two independent experiments only.

Downregulated Pathways by miRNA transfection Compared with the UNC miRNA			
	Pathway Name	Adj. *p*-value	Contributing genes
miR-100* vs UNC	-	-	-
miR-71 vs UNC	Interferon Gamma Response	<0.01	*IFITM3, EIF4E3, IRF4, PSMA2*
	IL-2/STAT5 Signaling	0.04	*IFITM3, IRF4, CD48*
	mTORC1 Signaling	0.04	*EBP, TFRC, CCNG1*
miR-10021 vs UNC	-	-	-
Combo* vs UNC	Interferon Alpha Response	0.07	*LGALS3BP, IFIH1, LY6E*
miR-1* vs UNC	Protein Secretion	0.06	*AP2S1, VPS45, SEC24D*
	Oxidative Phosphorylation	0.06	*UQCRQ, MRPS11, MRPS12, ATP6V1D*
	mTORC1 Signaling	0.06	*SLC9A3R1, SQSTM1, ATP6V1D, NFKBIB*
	Myc Targets V1	0.06	*PSMA2, RUVBL2, NHP2, SNRPA*
Transfection reagent vs UNC	IL-2/STAT5 Signaling	<0.01	*PENK, LRRC8C, PIM1, LIF, CD48*
	mTORC1 Signaling	0.01	*TFRC, DHCR24, TMEM97, ATP6V1D*
	Inflammatory Response	0.01	*OSM, LIF, LTA, CD48*
	Fatty Acid Metabolism	0.04	*GPD2, FASN, DHCR24*
**Upregulated pathways by miRNA transfection compared with the UNC miRNA**			
-	**Pathway name**	**Adj. p-value**	**Contributing genes**
Transfection reagent vs UNC	TNF-alpha signaling *via* NF-kB	<0.01	*NR4A1, BTG2, EGR2, IRF1, PDE4B, MXD1*
	Apoptosis	0.01	*TGFBR3, BTG2, CASP7, BCL2L11, IRF1*
	Interferon Gamma Response	0.01	*CASP7, IRF1, PDE4B, JAK2, SAMHD1*
	Complement	0.01	*CASP7, SRC, BRPF3, IRF1, JAK2*
	UV Response Up	0.03	*NR4A1, BTG2, BCL2L11, IRF1*
	IL-2/STAT5 Signaling	0.04	*CTLA4, CAPN3, MXD1, IGF1R*
	Inflammatory Response	0.04	*BTG2, IRF1, PDE4B, MXD1*
	heme Metabolism	0.04	*BTG2, CIR1, TENT5C, KDM7A*

A closer look at the most strongly up- and downregulated genes provides further insights into the effects of nematode miRNA mimics on expression of individual genes. SpiB, a transcription factor associated with the DN3 stage in T cells, was among genes with the highest fold change after transfection with miR-71, miR-100, and miR-10021 individually or in combination. Among the most strongly downregulated genes, a large fraction shows involvement in cell metabolism, with no unique functions in T lymphocytes. Determinants of lysosomal function (e.g., transcription factor EB (*Tfeb*) and N-sulfoglucosamine sulfohydrolase (*Sgsh*)) were downregulated. Of special interest was the downregulation of interferon-inducible transmembrane (IFITM) proteins (*Ifitm3*) and homeodomain-only protein homeobox (*Hopx*), both associated with CD4^+^ T cells differentiation potential (Opejin et al., 2020; Yánez et al., 2019, 2020). CD48 is crucial for CD4^+^ T cell activation, as observed in CD48-deficient mice (González-Cabrero et al., 1999); however, downregulation by miR-71 is likely non-specific since *Cd48* expression was decreased by the transfection reagent alone. The mTOR signaling controls multiple effector T cell fates, including Th2. mTORC1 regulates exit from quiescence of naïve T cells, which undergo metabolic reprogramming (Yang et al., 2013). For instance, mTORC1 activates transcription factors (e.g., HIF-1α and MYC), which in turn stimulate transcription of the transferrin receptor (TFRC; downregulated upon miR-71 treatment), required to import cofactor Fe^3+^ to support cell growth and proliferation (Truillet et al., 2017). Among molecules of the transcriptional network and positive regulation of Th2 lymphocyte differentiation (Figure 1), our transcriptome analysis did not confirm significant regulation of *Gata3, Il4ra, Nfat, NFkB,* or *c-Maf.* However, interferon regulatory factor 4 (*Irf4*) showed a modest but significant decrease (log2 FC = -0.19) in miR-71 exposed cells. IRF4 promotes differentiation of naïve CD4^+^ T cells into Th2, Th9, Th17, or T follicular helper cells. It is required for functional effector regulatory T cells, as well as for the differentiation of many myeloid, lymphoid, and dendritic cells (Huber and Lohoff, 2014). Several mechanisms exist: IRF4 may promote IL-4 production by binding to the IL-4 promoter directly and in cooperation with members of the NFAT transcription factor family, by indirectly regulating IL-2-mediated Th2 expansion, or by upregulating GATA3 expression. In fact, overexpression of GATA3 may rescue IL-4 production in *Irf4* ^−/−^ Th2 cells, suggesting a pivotal role for IRF4-dependent GATA3 expression for T cell differentiation into Th2 (Lohoff et al., 2002; Huber and Lohoff, 2014). IRF4 expression is primarily induced by antigen receptor engagement, stimulation with LPS, or signaling induced by CD40 or IL-4 (Gupta et al., 1999). In T cells, its expression declines after reaching peaking levels within a few hours following TCR stimulation, when cells return to a resting state (Huber and Lohoff, 2014). IRF4 also likely acts in conjunction with BATF and JUN. Finally, IL-2—STAT5 signaling is among pathways tuned down by miR-71, *via* downregulation of several genes, which is another key element of the transcriptional network for Th2 cell differentiation.

### Nematode miRNAs Interact Directly With Predicted Binding Sites on Host Genes

**FIGURE 4:**
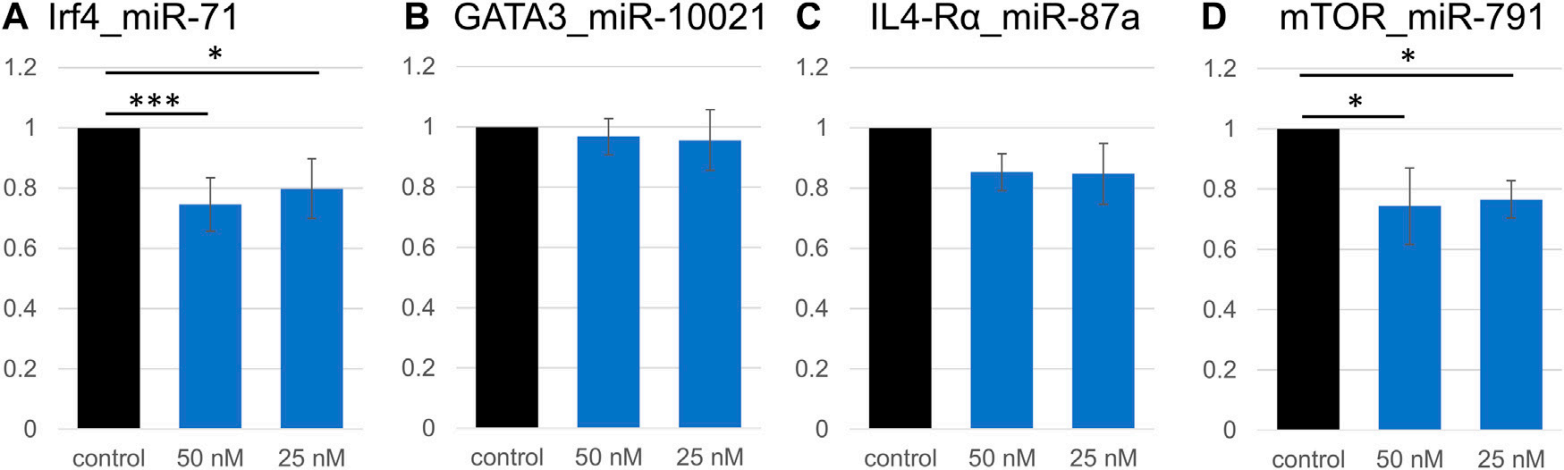
Validation of direct interactions between miRNAs and predicted targets. Relative luciferase activity is significantly decreased due to the insertion of a miR-71 binding site on *Irf4* **(A)**. Signal repression was stronger at 50 nM miRNA (*p* < 0.001, *t*-test) than at 25 nM (*p* = 0.02). Similarly, 50 nM or 25 nM miR-791 led to a decrease in luminescence signal (*p* = 0.02 and *p* = 0.03, respectively; **(D)**, confirming an interaction between miR-791 and its predicted binding site in *Mtor*. No significant decrease in luminescence was detected in all other miRNA:binding site combinations (*Gata3*:miR-10021 **(B)**, and *Il4ra*:miR-87a **(C)**). *p < 0.05, ***p < 0.01.

Because of expected compensatory regulatory mechanisms and potential indirect effects in cell systems, we investigated the accuracy of some computationally predicted targets. Four genes were selected from the Th2 transcriptional network, and from the downregulated pathways identified by RNA sequencing. Predicted binding sites for several miRNAs in *Gata3*, *Irf4*, *Mtor*, *Il4ra*, and Arginase 1 (*Arg1*) were cloned into the pmirGLO dual luciferase vector (Supplementary Table S4). These genes present multiple miRNA binding sites; miRNAs with the most promising computed context scores (TargetScan) were selected for validation. We validated that a direct interaction occurs between the cloned predicted miR-71 binding site on the *Irf4* mRNA, which supports results from the transcriptome analysis; exposure to 50 nM miR-71 (+ transfection reagent) led to a 25% reduction in luminescence, while 25 nM led to a 20% reduction (Figure 4). Similarly, the interaction between *Mtor* and miR-791 was confirmed, 50 and 25 nM miRNA leading to reduction of the relative Luciferase activity by 22 and 20%, respectively. In both cases, there was a very mild concentration dependence, in which the higher miRNA concentrations had a slightly more pronounced effect. The minor difference between both concentrations likely suggests saturation of the system, where only one binding site was cloned into the luciferase plasmid. We could not confirm an interaction between *Gata3* and miR-10021 or miR-71 (relative luciferase activity unchanged, not shown). miR-87a was found to be weakly interact with *Il4ra*, which did not reach significance levels. miR-71 did not affect luminescence signals when its most likely binding site on *Il4ra* was inserted into the vector. There was no identifiable interaction between miR-100 and its predicted site on *Mtor* and on *Arg1*.

### miR-71 Impacts Macrophage Cytokine Expression

**FIGURE 5:**
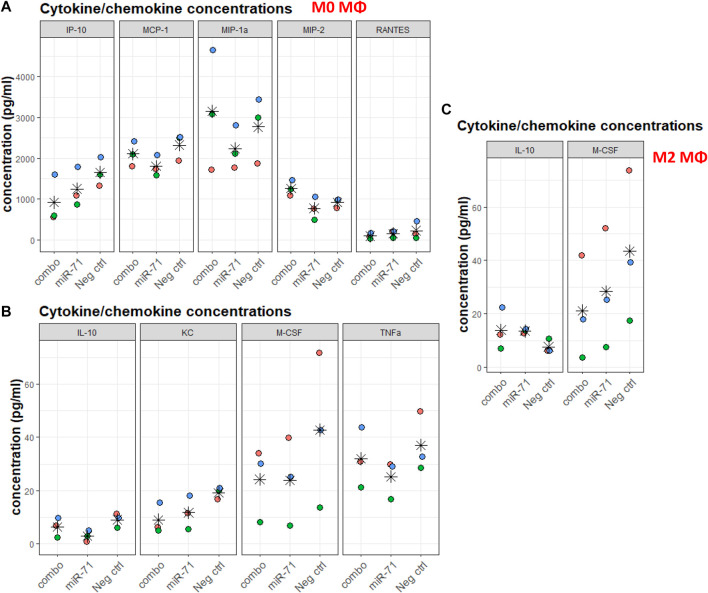
Cytokine/chemokine concentrations in supernatants of macrophages exposed to nematode miRNAs. For each analyte, colors indicate replicates, stars indicate the mean across three data points. **(A,B)** Chemokine/cytokine concentration (in pg/ml) varying upon miRNA transfection of M0 macrophages. **(C)** Chemokine/cytokine concentration (in pg/ml) varying upon miRNA transfection and polarization toward the alternative activation profile M2. IP-10: IFN-γ-induced protein 10 (Cxcl10); MCP-1: monocyte chemoattractant protein 1 (CCl2); MIP-1a: macrophage inhibitory protein 1a; MIP-2: macrophage inhibitory protein 2; RANTES: regulated upon activation, normal T cell expressed and presumably secreted (CCl5); IL-10: interleukin 10; KC: keratinocytes-derived chemokine (CxCl1); M-CSF: macrophage colony-stimulating factor (Csf1); TNF-α: tumor necrosis factor 1α. All concentrations were within range of detection 3.2–10,000 pg/ml (and 12.8–40,000 pg/ml for IL-13). Statistical significance of variation across conditions was performed against Neg. ctrl (UNC) using the Dunnett’s test.

Helminths and helminth EVs influence macrophages. The individual capacity of miRNAs (as opposed to whole EVs, containing many other molecules) to modulate the capacity of macrophages to differentiate was assessed. Without further stimuli, Raw 264.7 macrophages are unpolarized (M0). Cells were transfected with miR-71 alone or combined with miR-100 and miR-10021 (combo), or with the UNC; 24 h later, polarization was initiated either toward the classical activation profile (M1) or the alternative activation profile, associated with Th2 responses (M2). Supernatants were collected after 6 h for multiplex cytokine/chemokine analysis. Among the 32 measured analytes, most variation was observed in the unpolarized M0 population. Despite substantial variation across three biological replicates, the direction of regulation is reproducible across independent experiments and thus, lends some confidence in the data, although based on a tiny sample size, that did not reach significance. While more samples will be necessary to obtain fully conclusive results, in most cases, miR-71 showed an inhibitory effect by trend, compared to the UNC. IP-10 (CxCl10), MCP-1 (CCl2), MIP-1a, MIP-2, RANTES (CCl5) (Figure 5A), IL-10, KC (CxCl1), M-CSF (Csf1), and TNF-α, showed slight reductions in concentration (Figure 5B). The picture is not so clear regarding the effects produced by transfection with the three-miRNA combination, suggesting that the other two miRNAs (miR-100 and miR-10021) do not have the same effect as miR-71. It also shows concentration-dependence, as miR-71 is diluted 1:3 in the combo treatment, unless the other miRNAs have a stimulatory effect on cytokine production. Cytokine/chemokine concentrations produced by M1 macrophages were not affected by miR-71 or the miRNA combination compared to the UNC. In M2 supernatants, only IL-10 and M-CSF showed altered levels compared to the UNC, however, with large inter-replicate variation for M-CSF (Figure 5C). In M2 macrophages, IL-10 was mildly increased upon prior exposure to miR-71, which would support an active regulatory role of this helminth miRNA by acting on innate immune cells in type 2 immune responses. IP-10 is predicted to be targeted by miR-100 (see Supplementary Table S4 and target predictions: https://data.mendeley.com//datasets/358nm9mhpr/1). miR-71 is predicted to target transcripts of MIP-1a, KC, M-CSF *via* 7mer-1 or 7mer-8 binding sites. miR-1 is predicted to target MIP-1a and M-CSF *via* both kinds of 7mer sites as well. Similarly, miR-10021 is predicted to bind to MIP and M-CSF, while miR-100 is predicted to interact with IP-10. TNF-α, MCP-1, and RANTES were not among putative targets of the miRNAs we used in transfection experiments.

## Discussion

Parasitic helminths are large eukaryotic pathogens with complex life histories that tend to persist for relatively long periods of time in their hosts. Through the release of molecular messages in their surroundings, they master the art of host manipulation. A contribution of EVs in interspecific crosstalk has been increasingly documented. However, EVs represent inhomogeneous entities showing a great molecular diversity derived from parent cells. It is difficult to assign an observed effect on host cells to a specific molecule in this case. Differentiating helminth products that are important for infection from those that are not involved in the ‘negotiations’ between hosts and parasites will likely require a thorough breakdown of the molecular spectrum constituting E/S products (ESP), using models that can reduce the complexity of host-parasite communication.

Previously, *H. polygyrus bakeri* EVs were shown to have various effects on MODE-K cells (an intestinal cell line) (Buck et al., 2014). The most strongly downregulated gene was *Dusp1* (dual-specificity phosphatase 1), a regulator of MAPK signaling associated with dampening the type 1 pro-inflammatory responses. Another gene significantly downregulated by exosomes is *Il1rl1* (also known as IL33R in humans), the ligand-specific subunit of the receptor for IL-33, key in responses to helminths (Buck et al., 2014; Coakley et al., 2017).

*Heligmosomoides polygyrus bakeri*, *T. muris* and *A. suum* are commonly used as models of human intestinal infections. They infect laboratory mice for at least a part of their life cycle. Previous studies suggested macrophages and T cells as particularly exposed to nematode secreted miRNAs and predicted a functional impairment (Quintana et al., 2019; Duguet et al., 2020; Meningher et al., 2020). In this work, a small fraction of the miRNA complement of helminth ESP was examined for host gene regulatory properties in cells of the innate and adaptive arms of immunity. Helminths typically elicit a Th2 effector cell polarization, along with the induction of a prominent regulatory T cell (Treg) population. To assess whether nematode miRNAs are indeed capable of modulating T cell differentiation or proliferation, we relied on a simplistic conditioning *in vitro* model (without reaching the fully differentiated state), that allowed for manipulation but has a limited ability to reflect the complexity and intercellular interactions required to produce an immune response *in vivo*. Moreover, despite our effort to expose cells repeatedly, our assay setup likely did not accurately mirror the constant exposure of local immune cells to helminth products. miRNA overexpression studies have shown that cells start to recover normal gene expression levels as soon as 12 h post-transfection, indicating that effects are transient (Jin et al., 2015). The combination of low miRNA concentrations and punctate transfections may have contributed to the observation of only a small number of DE genes in the transcriptome analysis. Nevertheless, the approach identified IRF4 (also known as NF-EM5, PIP, MUM1, or ICSAT), a crucial and direct interactor of GATA3, the master regulator of Th2 differentiation, as a target of one of the most abundantly secreted nematode miRNAs. Repression observed in reporter assays was in the range of prior observations with nematode miRNAs (Buck et al., 2014; Meningher et al., 2020). Of note, dendritic cells (DCs) expressing IRF4 (IRF4+ CD11c+ CD11b+ DCs) are potent drivers of type 2 immunity in *T. muris* infection, while other DC populations are associated with establishment of chronic infections (Mayer et al., 2017; Else et al., 2020). *T. muris*, however, does not express miR-71, and hence, does not rely on this particular modulatory mechanism (Sotillo et al., 2020; White et al., 2020). In macrophages too, the mTORC2-IRF4 signaling axis is essential for alternative activation (M2), a profile observed upon *H. polygyrus bakeri* infection. Hence, given the importance of IRF4 at several levels in the anti-helminth immune response(s), modulation of this gene is expected to have functional consequences. This will require verifications, especially in additional cell types such as DCs or macrophages, given their prominent role in responses to nematode infections.

To validate additional and potentially crucial nematode miRNA targets in Th2 cell differentiation, we selected a number of miRNA:mRNA sites for closer examination using reporter assays. We confirmed an interaction between miR-791 and its binding site on *Mtor*. Th2 cell differentiation requires mTORC2 activity (Delgoffe et al., 2011). In addition, the mTORC1-mediated pathway links signal-dependent metabolic reprogramming (through CD28 or TCR) to exit the quiescent state, and subsequently orchestrate cell proliferation and fate decisions. Together with Raptor, the mTORC1 complex connects glucose metabolism to cytokine responsiveness and Th2 cell differentiation and expansion (Yang et al., 2013). EVs released from the filarial nematode *Brugia malayi,* enriched for miR-100, miR-71, miR-34, and miR-7, downregulate the host mTOR pathway as well (Ricciardi et al., 2021). Their target predictions agreed with ours, in that miR-100 was predicted to target mTOR, among other mRNAs (e.g., eukaryotic translation initiation factor 4E binding protein 1 (EIF4E3BP1)) in the mTOR signaling pathway. EIF4E3BP1 was not identified among the targets of miRNAs from our nematode species, but *Eif4e3* was among downregulated genes following transfection with miR-71, according to transcriptomics data. Recent evidence shows that EIF4E3 acts in re-programming the translatome to promote ‘stress resistance’ and adaptation and is recruited the mTOR pathway is inhibited (Weiss et al., 2021). A further gene downregulated by miR-71 treatment was *Ifitm3*, which is constitutively expressed in CD4^+^ T cells and is downregulated upon cell activation. In a mouse model and in contrast to *Ifitm2*, *Ifitm3* deletion did not reduce the Th2 response; hence, its role in a context of cell polarization remains incompletely understood (Yánez et al., 2019). *Ifitm3* was not among direct targets of the transfected miRNAs, hence, the effect is likely indirect.

Raw 264.7 macrophages transfected with miR-71 or the miRNA combo (miR-71, miR-100, miR-10021 combined) resulted in mild alterations of mRNAs encoding some cytokines/chemokines. Especially in non-polarized cells, cytokine levels were low at baseline and differences subtle. Although not statistically significant and based on limited sample sizes, miR-71 exposure led to systematically decreased analyte levels by trend, compared to UNC treatment, and the direction of regulation was reproducible across independent experiments. In both non-polarized (M0) and alternatively activated macrophages, miR-71 and the miRNA combination decreased macrophage colony-stimulating factor (M-CSF) levels. M-CSF is required for macrophage differentiation. Lowered M-CSF levels might lead to impairment of the innate immune response and weakened macrophage capacity to respond appropriately to worm infection. While Zamanian and colleagues reported substantial increases in monocyte chemoattractant protein (MCP-1), macrophage inhibitory protein 2 (MIP-2), RANTES, and TNF-α following uptake of *B. malayi* larvae EVs, in line with the classical activation profile, we observed the opposite upon introduction of miR-71 into cells. EVs, however, represent more complex entities than just a handful of miRNAs, and EV composition (including miRNAs) tend to vary in a species- and stage-specific manner (Zamanian et al., 2015; Sotillo et al., 2020). Decreases in IP-10 (IFN-γ-induced protein, or CxCl10), MIP-1, and KC (CxCl1, a neutrophil chemoattractant), and increased levels of IL-10 (an anti-inflammatory cytokine) in M2 macrophages suggest that miR-71 (and to some extent combo, where miR-71 is “diluted” with other miRNAs) has potential both anti-inflammatory and immunosuppressing properties and would overall favor polarization toward the alternative activation profile.

In general, miRNAs are considered key mediators in development, activation and regulation of immune cells (Arora et al., 2017). In light of their redundant and pleiotropic properties, it is likely that only a handful of different miRNAs are not sufficient to produce a phenotype. Indeed, we did not observe any significant difference in terms of cell counts or Th2 proportions. miRNA-driven silencing is often relatively subtle: they are “micromanagers” contributing to of regulatory circuits and control fine-tune of protein expression (Arora et al., 2017). In addition, cells are expected to have many compensatory mechanisms to function when one pathway gets slowed down. Hence, it is likely that the cumulative effects of multiple different miRNAs may have more pronounced, synergistic effects on host cells.

In *C. elegans*, miR-71 promotes longevity and stress resistance (Boulias and Horvitz, 2012). In addition, miR-71 is specifically required for the starvation-induced stress response and plays a critical role in long-term survival by repressing the expression of insulin receptor/PI3K pathway genes and genes acting downstream or in parallel to the pathway (Zhang et al., 2011): mutating miR-71 drastically reduces the survival rate of animals in first stage larvae (L1) diapause (Zhang et al., 2011), a stress-resistant, developmentally quiescent, and long-lived larval stage. In *Echinococcus multilocularis*, miR-71 is required for metacestode normal development *in vitro* (Pérez et al., 2019). Taken together and integrating data presented here, it is likely that miR-71 fulfills multiple roles, both endogenously and exogenously.

Only few predicted targets have translated into validated targets using reporter assays. Generally, the most effective canonical site types are the 8mer sites, where a Watson–Crick match to mature miRNA positions two to eight with an A opposite position 1 is observed (Lewis et al., 2005; Agarwal et al., 2015), followed by 7mer sites. Finally, 6mer site types are associated with weaker preferential conservation and much lower efficacy (Friedman et al., 2009; Agarwal et al., 2015). In line with this, the predicted 6mer sites on IL4-Rα and GATA3 upon interaction with miR-71 resulted in no decrease in luminescence in reporter assays. Binding sites between miR-71 and IRF4 and mTOR and miR-791 were 8mer-1a, and 7mer-m8, respectively. Despite optimal site conservation, GATA3 and IL-4Rα are not confirmed as biochemical targets of miR-10021 (8mer-1a) and miR-87a (7mer-m8), respectively. Therefore, predicted targets, even with high context scores and optimal site conservation parameters, require experimental validation.

The fact that a nematode-specific miRNA, without a close mammalian homologue, may incorporate into host AGO protein complexes support the hypothesis that the silencing machinery does not need to be transferred along with small RNAs (Donnelly and Tran, 2021). miR-71 was not detected in mesenteric lymph nodes of *H. polygyrus bakeri* infected mice, likely present below the detection level of the assay. Meningher et al. reported measurable amounts of miR-10 and Bantam from the mesenteric lymph nodes and Peyer’s patches of *S. mansoni* infected mice, but not in the inguinal lymph node nor in the spleen (Meningher et al., 2020); adult worms reside in the mesenteric and small intestine venules and hence, have a more direct contact with lymphoid tissue surrounding the parasite, than would do the gastrointestinal nematodes.

Whether nematode miRNA concentrations achieve biologically relevant *in vivo* (for local and systemic effects) is currently under investigation in our laboratory. However, the low miRNA doses (10 nM for 2 × 10^5^ to 1 × 10^6^ cells) we used in the present work support the hypothesis that they might indeed at least have mild but reliable local regulatory effects.

This work demonstrates that repeated exposure to low concentrations of lipid-encased synthetic nematode miRNAs can result in a mild modulation of host gene expression *in vitro*, which was partially confirmed by reporter assays. In particular, abundant GI nematode miRNA sequences may get loaded onto host AGO proteins and target important components of the Th2 differentiation process: IRF4 and mTOR. Immunomodulatory effects exerted by individual parasite miRNAs are very mild in comparison to whole worms or EVs but are in line with their proposed roles as fine-tuning entities of gene and protein expression. Further mechanistic insights are required in order to fully grasp the extent of miRNA-mediated host modulation.

## Data Availability

The datasets presented in this study can be found in online repositories. The names of the repository/repositories and accession number(s) can be found below: https://data.mendeley.com//datasets/358nm9mhpr/1, 10.17632/358nm9mhpr.1, https://www.ncbi.nlm.nih.gov/geo/, GSE180829.
